# The Role of the Lymphatic System in the Pathogenesis and Treatment of Inflammatory Bowel Disease

**DOI:** 10.3390/ijms23031854

**Published:** 2022-02-06

**Authors:** Dimitrios Nikolakis, Floris A. E. de Voogd, Maarten J. Pruijt, Joep Grootjans, Marleen G. van de Sande, Geert R. D’Haens

**Affiliations:** 1 Department of Gastroenterology, Amsterdam Institute for Gastroenterology Endocrinology and Metabolism, Academic Medical Center, Amsterdam UMC, University of Amsterdam, Meibergdreef 9, 1105 AZ Amsterdam, The Netherlands; d.nikolakis@amsterdamumc.nl (D.N.); f.a.devoogd@amsterdamumc.nl (F.A.E.d.V.); m.j.pruijt@amsterdamumc.nl (M.J.P.); j.grootjans@amsterdamumc.nl (J.G.); 2 Department of Rheumatology and Clinical Immunology, Amsterdam Rheumatology & Immunology Center (ARC), Amsterdam UMC, University of Amsterdam, Meibergdreef 9, 1105 AZ Amsterdam, The Netherlands; m.g.vandesande@amsterdamumc.nl; 3 Department of Experimental Immunology, Amsterdam Institute for Infection & Immunity, Amsterdam UMC, University of Amsterdam, Meibergdreef 9, 1105 AZ Amsterdam, The Netherlands; 4 Onassis Foundation, 4 Aeschinou Street, 10558 Athens, Greece

**Keywords:** IBD, lymph nodes, Peyer’s patches, stroma, lymphatic obstruction, microbiome, lymphocyte trafficking

## Abstract

Although the number of therapeutic options for the treatment of inflammatory bowel disease (IBD) has increased in recent years, patients suffer from decreased quality of life due to non-response or loss of response to the currently available treatments. An increased understanding of the disease’s etiology could provide novel insights for treatment strategies in IBD. Lymphatic system components are generally linked to immune responses and presumably related to inflammatory diseases pathophysiology. This review aims to summarize findings on immune-mediated mechanisms in lymphoid tissues linked with IBD pathogenesis and (potential) novel treatments. Enhanced innate and adaptive immune responses were observed in mesenteric lymph nodes (MLNs) and other lymphoid structures, such as Peyer’s patches, in patients with IBD and in animal models. Furthermore, the phenomenon of lymphatic obstruction in the form of granulomas in MLNs and lymphatic vessels correlates with disease activity. There is also evidence that abnormalities in the lymphatic stromal components and lymph node microbiome are common in IBD and could be exploited therapeutically. Finally, novel agents targeting lymphocyte trafficking have been added to the treatment armamentarium in the field of IBD. Overall, gut-associated lymphoid tissue plays a key role in IBD immunopathogenesis, which could offer novel therapeutic targets.

## 1. Introduction

Inflammatory bowel diseases (IBD) are chronic immune diseases of the gut, with Crohn’s disease (CD) and ulcerative colitis (UC) being the two most common phenotypes. The pathophysiology of IBD encompasses a complex interplay of the adaptive and innate immune system, the microbiome, genetic susceptibility, and environmental factors, but the exact etiology remains unknown. The cornerstone of the treatment for both CD and UC is targeting pro-inflammatory pathways. Although the increased number of treatment options over the past decades has led to important progress, many patients are non-responders or experience loss of response, resulting in a relapse of the disease, damage to the gastrointestinal tract and long-term complications [1]. In order to progress towards better treatment options and personalized medicine in IBD, more knowledge regarding its etiology and the underlying immune-mediated mechanisms driving the disease pathogenesis is mandatory.

Over recent decades, there is increasing evidence for the involvement of lymphoid tissues in the pathogenesis of IBD. For example, lymphadenopathy is a frequent finding in patients with IBD, predominantly characterized by the increased number and size of mesenteric lymph nodes (MLNs) [2,3,4,5]. In addition, B and T-lymphocyte trafficking between the gut-associated lymphoid tissue (GALT) and the intestinal mucosa, induced by lymphocyte–dendritic cell interaction, is linked to the onset of IBD [6,7]. Moreover, lymphatic vessel functional and structural abnormalities have been observed in IBD [8,9,10]. Lastly, the luminal microbiota also colonizes lymphoid tissues and may thereby trigger further immune responses [11,12]. Consequently, targeting lymphocyte trafficking between lymph nodes and the intestinal mucosa is a potentially effective treatment in IBD [13,14].

Here, we reviewed the currently available knowledge on the involvement of the lymphatic system in the pathogenesis of IBD, and in novel (potential) treatment targets.

## 2. Results

### 2.1. The Involvement of the Lymph Nodes in IBD

#### 2.1.1. Innate Immune Responses

##### The Role of Mesenteric Lymph Node Dendritic Cells

Dendritic cells (DCs) in the lamina propria are important for homeostasis in the gut. They are involved in the transport of dietary and microbial antigens to the mesenteric lymph nodes (MLNs), in order to induce oral tolerance to nutrients and commensal gut bacteria by skewing T cells towards a regulatory phenotype [15,16].

In patients with IBD (UC and CD), both myeloid and plasmacytoid DC subsets have been identified in MLNs [17] and there is evidence showing that they are involved in the disease pathophysiology by inducing Th1/ Th17 responses.

It has been shown in rats with trinitrobenzene sulfonic acid (TNBS)-induced colitis that MLN myeloid (conventional) DCs have been involved in driving pro-inflammatory responses by polarizing T helper cells into a pro-inflammatory Th1 subset through interleukin (IL)-12 [18]. Furthermore, in patients with IBD (UC and CD), MLN myeloid DCs induce Th1 responses by the production of IL-12 and IL-23 [19].

In addition, it has been demonstrated in mice with dextran sulfate sodium (DSS)-induced colitis that MLN CD103^−^ DCs overexpressing the cytokine osteopontin induce Th1 and Th17 polarization and can exacerbate the disease. Interestingly, osteopontin-induced Th1 and Th17 polarization was required for disease initiation, since antibody-blocking of osteopontin made mice less susceptible to colitis by reducing MLN CD103^−^ DCs and Th1/Th17 cells, as well as by increasing MLN regulatory T-cell (T-regs) numbers [20]. Similar observations were made for the CD172a^+^ CD103^−^ DC subset. CD172a is a regulatory membrane glycoprotein called signal regulatory protein a (SIRPa) and is expressed in cells of myeloid origin. Increased levels of CD172a^+^ CD103^−^ DCs were observed in the MLNs and inflamed mucosa in TNBS colitis patients and mice with CD. These DCs are implicated in neutrophil migration, phagocytosis, as well as Th17 polarization, which was associated with increased CD47 glycoprotein receptor expression, leading to disease onset [21,22]. Other subsets of CD172a^+^ DCs expressing a specific glucosaminoglucan (CD172a^+^6-sulfo LacNAc DCs) have been observed in higher numbers in the MLNs of patients with CD compared to non-IBD controls, but their exact function in human IBD needs further elucidation [23].

It has been also observed that tolerogenic MLN DCs, characterized by expression of CD103, are diminished in patients with UC [24]. CD103^+^ DCs display their anti-inflammatory properties by inducing T-reg cell differentiation [25]. In a CD4^+^CD45RB^hi^ T cell adoptive transfer mouse model of colitis, it has been shown that these MLN DC subsets lose their regulatory function and acquire a more proinflammatory phenotype [26]. Complementary to these studies, two distinct populations of myeloid DCs expressing HLA-DR in high levels and intermediate levels were identified in patients with IBD. HLA-DR expression was shown to be inversely correlated with intestinal inflammation, since it was lower in DCs of MLNs draining inflamed colon, compared to MLNs draining non-inflamed colonic tissue, but the exact function of these myeloid DC subsets is not clear [27]. In contrast to the decreased numbers of the CD103^+^ myeloid tolerogenic DCs in MLNs, tolerogenic plasmacytoid DCs (pDCs) have been shown to be increased in MLNs of patients with IBD, but these pDCs do not seem to alter disease progression [28,29].

Further findings in colitis animal models suggest that the TGF beta pathway in DCs plays an important role in regulating the dysbalance between proinflammatory T-cells and T-regs in MLNs. TGF-beta-1 overexpression in non-differentiated DCs of DSS colitis mice upregulates T-regs in MLNs that delay the disease progression, whereas in differentiated DCs it promotes effector T-cell trafficking from MLNs to the colonic mucosa by upregulating the expression of the gut homing integrin α4β7, leading to colitis induction [30,31].

In summary, these findings show that specific pro-inflammatory DC subsets are increased in the MLNs in the setting of colonic inflammation, and these subsets are involved in driving Th1 and Th17 responses, which are both important pro-inflammatory T cell subsets involved in IBD pathogenesis [32,33,34,35]. Additionally, some tolerogenic DC subsets associated with T-reg differentiation and tolerance induction are decreased, which could further contribute to inflammation [24,26]. Overall, these studies provide initial evidence that MLN DCs can shape the balance between proinflammatory and regulatory responses, which shifts towards inflammation in IBD.

##### The Role of Mesenteric Lymph Node Macrophages, Mononuclear Phagocytes and Basophils

Macrophages in the intestinal mucosa lamina propria clear microbiota and produce both pro- and anti-inflammatory cytokines [16]. These cells are thought to play a pivotal role in IBD pathogenesis. In the LNs, macrophages may also contribute to the disease pathogenesis, for example, by inducing pathogenic T cell responses. Indeed, CD64^+^ macrophages accumulated in MLNs in a T-cell-mediated colitis mouse model and they induced Th1 polarization in vitro when co-cultured with naïve T-cells [36].

Macrophages expressing the adhesion protein CD169 (Sialoadhesin) constitute a population that is distinct from the well-known pro-inflammatory M1-like and anti-inflammatory M2-like macrophages. This CD169^+^ macrophage subset is located in secondary lymphoid organs and can interact directly with B- and T-lymphocytes by presenting (microbial) antigens [37,38]. A CD11b^+^ CD169^+^ macrophage subset was found to be significantly increased in MLNs in the acute phase of mice with DSS-induced colitis and was associated with pathogenic Th17 responses [39]. Human studies investigating this macrophage population are lacking.

What is known in human IBD is that MLN HLA-DR^+^ SIRPa^+^ DC-like mononuclear phagocytes drive the emergence of a dual Th1/Th17 phenotype in MLN central memory T-cells, while both DC-like and macrophage-like mononuclear phagocytes amplify the MLN Th17 responses [40]. Moreover, increased numbers of basophil populations were accompanied by an elevation in Th1 and Th17 responses in MLN effector memory and central memory T-cells isolated from patients with IBD, as shown by in vitro experiments [41].

These studies further support that innate immune cells in MLNs can induce proinflammatory adaptive immune responses linked to intestinal inflammation in IBD.

#### 2.1.2. Adaptive Immune Responses

##### Mesenteric Lymph Node T-Cell Subtypes Alterations

Increased frequencies of highly proliferating lymphocytes have been observed in MLNs and lymphoid follicles from the inflamed intestine of patients with IBD, compared to non IBD controls [42,43]. The subsets of T cells differ when comparing CD with UC MLNs. For instance, patients with CD have higher frequencies of MLN Th17 cells with a pro-inflammatory/cytotoxic phenotype (IL-23,IL-22, granzyme B expression), whereas Th17 cells in the MLN of patients with UC have a more tolerogenic profile (CTLA-4,IL-10,FOXP-3 expression). Irrespective of their phenotype, however, IL-12 leads to a polarization of the Th17 cells into IFNγ expressing pathogenic Th1 cells [44].

The importance of both Th1 and Th17 subsets within the MLN was demonstrated in murine models [45,46,47,48,49,50,51]. The therapeutic efficacy (clinical and histological improvement of the disease) of various compounds, such as mTOR, AMPK kinase inhibitors, a RORγτ receptor inverse agonist, vitamin D3 and a peptide-based therapeutic vaccine targeting the p-40 subunit of IL-12/IL-23, has been associated with a significant reduction in Th1 and Th17 cell numbers in MLNs, accompanied by decreased pro-inflammatory IL-17 levels in the colonic mucosa, as well as by clinical and histological improvement in the disease [45,46,47,48,49,50,51]. These results indicate that MLN Th1/Th17 responses are key mediators in IBD progression.

In addition to Th1/Th17 T-cell subsets, T follicular helper (Tfh) cells may also be involved in abnormal immune responses observed in IBD. Tfh can both function as inflammatory effector cells and as regulatory cells. Tfh frequencies have been shown to be elevated in the germinal centers of MLNs in murine colitis models and disruption of Tfh differentiation by IRF-8 (interferon regulatory factor-8) downregulation can exacerbate the disease [52,53]. Furthermore, the inhibition of Tfh differentiation is associated with dysfunction of immune checkpoint molecules such as CTLA-4, in mice. CTLA-4 downregulation results in increased disease activity, characterized by humoral autoimmune-mediated intestinal damage [54,55]. These findings indicate that CTLA-4 and IRF-8 defects in Tfh cells might be implicated in the initiation of IBD.

Lymphocyte trafficking between the blood stream, gut and the lymphatic system plays an important role in the inflammatory process in IBD. Both in human IBD and animal models, increased numbers of T cells expressing adhesion molecules including L-selectin, V beta 8 integrin, α4β7 and VCAM-1 have been detected in MLNs [56,57,58,59]. Adhesion-molecule-expressing T cells can induce colitis when transferred to naïve mice [57]. Moreover, gut homing α4β7^+^CD4^+^T-cells is increased in the MLNs of immunodeficient mice transplanted with a gut wall graft from non-IBD immunocompetent mice, demonstrating that intestinal immune cells home to MLNs. These α4β7^+^CD4^+^T cells subsequently migrate to the intestine and induce IBD-like inflammation after their adoptive transfer to the immunodeficient mice [58].

Further evidence for the importance of T cell priming in MLNs is derived from murine and human studies, demonstrating that Th1 cells expressing CXCR3 (the receptor of the CXCL10 chemokine) can migrate to inflamed intestinal tissue [60,61]. Conversely, it has been suggested that impaired trafficking of anti-inflammatory cells such as T-regs can aggravate inflammation, as demonstrated in an adoptive T-lymphocyte transfer mouse model with IBD-like pathologies [62].

In conclusion, both increased effector T-lymphocyte trafficking and impaired T-reg trafficking from MLNs to inflamed intestinal tissue appear to play a role in IBD onset and the perpetuation of inflammation.

##### Mesenteric Lymph Node B-Cell Subtypes Alterations in IBD

The proportion of activated B lymphocytes in MLNs of patients with IBD is higher compared to peripheral blood or MLNs of healthy individuals [63,64,65]. There is also evidence of B-cell trafficking between the inflamed colon and draining LNs of patients with UC [66].

Animal studies indicate that humoral autoimmune responses in MLNs may play a key role in the initiation and progression of IBD. In T-cell receptor-a (TCR-a) knock out (KO) mice that spontaneously develop colitis, autoreactive B-cells producing anti-neutrophil cytoplasmic antibodies and antibodies against tropomyosin are present in MLNs, draining the diseased colon [67,68]. Tropomyosin is expressed on colonic epithelium, and anti-tropomyosin antibodies can damage colonic epithelial cells [69,70].

Specific MLN B-cell subtypes might also be important for immune tolerance. Adoptive transfer of the G-protein Galphai2-deficient CD4^+^T-cells induced spontaneous colitis in mice [71]. Galphai-2 protein is physiologically involved in the development of epithelial cell tight junctions [72]. It was shown that both B-cells and CD8a^+^ T-cells expressing Galphai-2 were required to prevent the onset of disease in the CD4^+^CD45RB^+^ T-cell transfer colitis model, but the precise mechanism behind this remains unclear [71]. In a TCR-aKO model of spontaneous colitis, it was also shown that MLN CD1d^+^IgM-producing B-cells could suppress intestinal inflammation through the production of IL-10 [73]. Conclusively, the tolerogenic properties of these MLN B lymphocytes correlated with reduced disease severity in these IBD animal models.

Thus far, no human studies have clarified whether MLN B cells have a more proinflammatory or tolerogenic phenotype in IBD.

**Figure 1:**
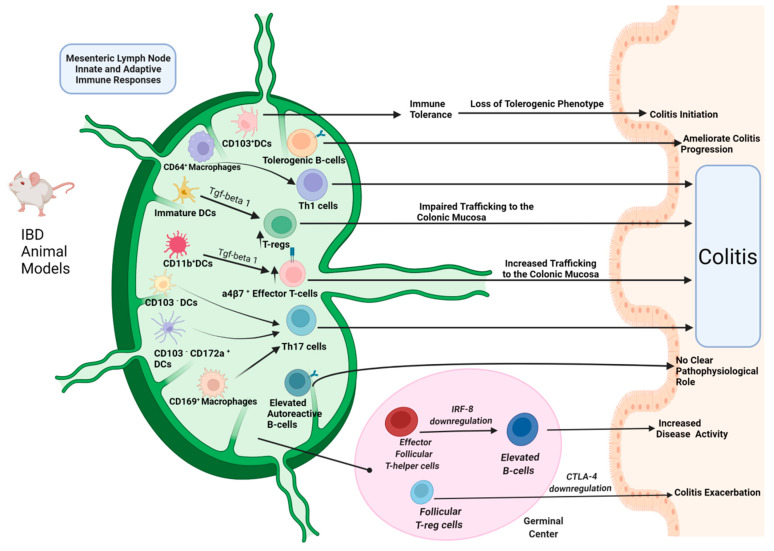
Adaptive and innate immune cell responses in mesenteric lymph nodes (MLNs) of IBD rodent models: Mature differentiated myeloid DC subsets orchestrate Th1 and Th17 inflammatory responses leading to disease onset, as well as, effector T-cell trafficking, whereas immature myeloid DCs induce immune tolerance by regulatory T-cell (T-reg) cell stimulation. Increased effector T-cell trafficking along with impaired T-reg trafficking to the colonic mucosa is associated with elevated inflammatory responses that can induce colitis. Tolerogenic MLN CD103^+^ DCs shift to a proinflammatory phenotype leading to colitis initiation, while MLN CD103^−^ DCs induce primarily Th17 lymphocyte polarization. Elevated macrophage subsets promote Th1 and Th17 differentiation in MLNs. Both Th1 and Th17 cells are responsible for the disease onset. Activation of effector follicular T-helper cells along with the downregulation of follicular T-regs leads to disease exacerbation. Elevated autoreactive B-lymphocytes can lead to humoral autoimmunity in colitis mice but with no clear function in the disease pathophysiology, while tolerogenic B-cells can attenuate the disease progression. (Figure created with BioRender.com-accessed date: 20 December 2021).

**Figure 2:**
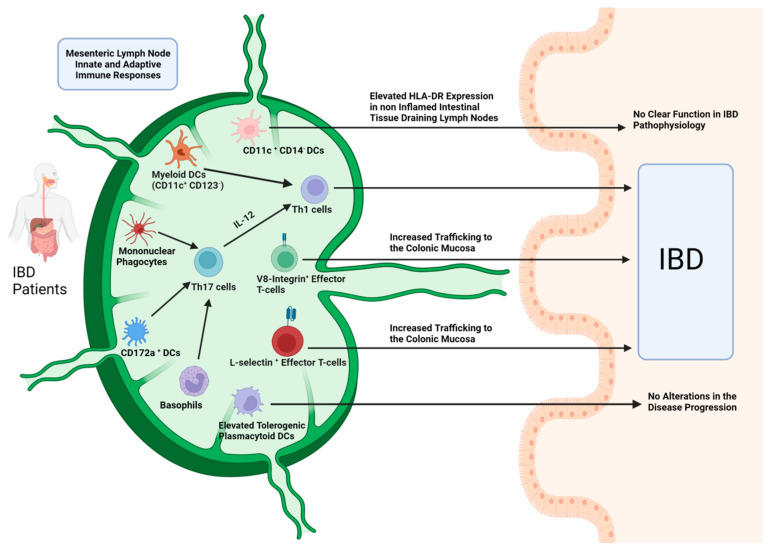
Adaptive and Innate Immune cell responses in MLNs of patients with IBD: Myeloid DC populations promote Th1 and Th17 responses linked with IBD initiation, whereas plasmacytoid DCs (pDCs) demonstrate a tolerogenic phenotype. However, these pDCs cannot alter the disease activity and progression. Basophils and mononuclear phagocytes promote Th17 responses. Th17 to Th1 polarization is a potential pathogenetic mechanism along with elevated effector T-cell trafficking to the colonic mucosa through lymphatic vessels. Reduced numbers of the tolerogenic CD103^+^ DCs in MLNs can further contribute to the disease pathogenesis. (Figure created with BioRender.com-accessed date: 20 December 2021).

In summary, all the above-mentioned human and animal IBD studies, provide substantial evidence for the involvement of both MLN innate and adaptive immune cell responses in the disease pathophysiology (Figure 1 and Figure 2).

### 2.2. The Involvement of Peyer’s Patches, and Colonic and Cecal Patches in IBD

It has been suggested that Peyer’s patches (PPs) play a role in IBD pathogenesis. Various innate and adaptive immune cells are present in PPs including conventional B and T cells, plasma cells, CD4^+^ TCR-β^+^ T-cells and γδ T-cells [74,75]. PPs have been shown to play an important role in the initiation of mucosal inflammation in patients with CD and in murine models, since bacteria, especially the adherent-invasive E.coli (AIEC) colonizing the PPs subepithelial dome, interact with resident DCs and, hence, acquire an inflammatory profile with elevated TLR-4 and TNF-a expression [76,77,78,79]. Th1 polarization has also been observed in the PPs of patients with active as compared to inactive CD, UC and healthy controls [80]. Furthermore, in mice with Galphai2 KO colitis, PPs contained increased numbers of apoptotic T- and Ig-producing B-lymphocytes, and this finding was associated with enhanced Th1 responses [81,82].

Overall, studies on the role of B cell populations in PPs are limited and both pro-inflammatory and anti-inflammatory B cell subsets have been described. For instance, increased numbers of memory B-cells were seen in the PPs and isolated lymphoid follicles of patients with IBD compared to healthy controls. Interestingly, these B cells had a proinflammatory phenotype, as demonstrated by the elevated expression of TNF-α [83]. In contrast, PPs in mice with DSS colitis contained more regulatory CD11b^+^ B-cells compared to healthy control mice, which had a protective effect on disease progression and displayed an IL-10-mediated immunosuppressive function that was dependent on hypoxia-inducible factor-a (HIF-1a) expression [84].

In addition to PPs, there is also an indication of the involvement of other lymphoid follicle structures in IBD manifestations. For instance, lymphoid nodules in the rectum that resemble PPs and contain elevated numbers of macrophages; CD4^+^ and CD8^+^ T-cells in mice with DSS-induced colitis, were linked with proximal disease extension [85]. On the other end of the colon, colitis mice with a cecal patch had an enhanced B-cell activation in these patches during the early phase of the disease [86]. In addition, these patches exhibited elevated Th2 responses associated with mucosal inflammation [87]. Whether the involvement of these lymphoid structures in immune deregulation leads to IBD needs further exploration in human studies to reach certain conclusions. These results introduce potential novel therapeutic interventions for IBD, such as an appendectomy with removal of the cecal patch. A case series of patients with UC undergoing appendectomy suggests a therapeutic benefit and we are awaiting the results of randomized controlled studies [88].

### 2.3. The Involvement of the Lymphatic System Stromal Components in IBD

#### 2.3.1. Lymph Node Stromal Cells and IBD

Studies investigating the role of lymph node stromal cells in IBD are scarce. In a murine model of intestinal autoimmunity, MLN stromal cells could partially promote T-cell tolerance by reducing only the CD8^+^ autoreactive T-cells [89]. Additionally, in mice with colitis, peripheral LN stromal cells were more tolerogenic and ameliorated the disease, compared to MLN stromal cells, which demonstrated a more proinflammatory profile [90].

#### 2.3.2. Adipose Tissue and Lymphatics in IBD: The Role of Perinodal Adipose Tissue

Abnormal interaction of the perinodal adipose tissue (PAT) and the lymphatic system might be related with IBD pathophysiology. The proportion of polyunsaturated fatty acids is greater in PAT of patients with CD compared to controls, whereas it was reduced in MLN lymphoid cells, indicating that perinodal adipocytes do not supply fatty acids to adjacent lymphoid cells, due to PAT defects in CD [91,92]. Furthermore, the perinodal mesenteric adipocytes of rats with TNBS-induced colitis produced elevated amounts of ω-6 polyunsaturated fatty acids, such as arachidonic acid, as well as increased levels of adipokines. The increased production of unsaturated fatty acids and adipokines from these perinodal adipocytes was associated with increased Th1 responses and cyclooxygenase-2 (COX-2) overexpression in the MLN T cells of diseased mice. These findings suggest that PAT alterations could contribute to lymph node T-cell activation during colitis [93,94]. Conclusively, deregulated PAT adipocyte responses might contribute to IBD through enhanced activation of MLN immune cells.

#### 2.3.3. The Role of Lymphatic Vessels and Lymphatic Endothelium Alterations in IBD Pathogenesis

The lymphatic vasculature is crucial for maintaining homeostasis. Proinflammatory mediators are drained and removed from the sites of inflammation through lymphatic vessels, whereas at the same time undifferentiated immune cells are directed to lymph nodes for their maturation [95]. Increased lymphangiongenesis and lymphangiectasia as well as elevated lymphangiogenic markers in lymphatic vessels have been observed in UC and CD in comparison to controls [8,9,96,97,98]. In mice with DSS-induced colitis, immunohistochemistry revealed that lymphangiogenesis has been associated with elevated numbers of DCs in MLNs, increased expression of prostaglandins in the lymphatic endothelium and with increased production of the proangiogenic factor VEGFC/D by intestinal macrophages. Therefore, these results link colitis-associated lymphangiogenesis with elevated inflammatory responses [99,100,101].

In a guinea pig TNBS ileitis model, dysfunction of lymphatic vessel contractility, as a consequence of increased hyperpolarization through the NO-KATP potassium channel, resulted in impaired lymphatic drainage and elevated inflammatory responses. The treatment of these mice with NO-KATP or COX inhibitors improved this contractility, suggesting that prostanoids affect lymphatic vessel contractile functions in intestinal inflammation [102,103]. Even though this has not been confirmed in humans, these studies indicate a correlation between inflammatory responses and lymphatic vessel abnormalities observed in IBD, but the exact mechanism underlying IBD onset and progression needs to be clarified.

The lymphatic vasculature plays an important role in lymphocyte trafficking. Both conventional lymphatic vessels and high endothelial venules (HEVs) contribute to an increased immune cell trafficking in IBD. HEVs are specific blood vessels that can be detected in secondary lymphoid organs or in tertiary lymphoid structures during inflammatory conditions. These vessels allow blood-circulating lymphocytes to enter the lymph nodes or inflamed tissues. It has been observed that HEVs are associated with increased infiltration of memory T-cells in the inflamed colonic mucosa of patients with IBD [104]. HEV endothelium in mice with colitis, as well as in patients with UC, is characterized by elevated expression levels of the adhesion molecules MadCAM-1 and the L-selectin-peripheral lymph node addressin, respectively [105,106], which can explain the T-cell trafficking between GALT compartments and the intestinal mucosa in IBD.

Interestingly, genetic polymorphisms of Vcam-1 expressed in lymphatic vessels, have been shown to be associated with increased disease susceptibility in IL-10KO colitis mice [107]. It was also shown that the mTOR-dependent expression of the IL-20RA cytokine in lymphatic vasculature regulates the circulation of lamina propria mononuclear cells (LPMCs) through human intestinal lymphatic endothelium in patients with CD [108]. This finding suggests that the mTOR signaling pathway could be a promising therapeutic target by interfering with immune cell trafficking.

Data on the exact relationship between lymphangiogenesis/lymphatic vessel density and disease activity are conflicting. In patients with CD that underwent ileocolonic resection (ICR), increased mesenteric lymphatic vessel density was associated with postoperative recurrence [109,110]. In other studies, decreased lymphatic vessel density, along with decreased levels of the lymphangiogenic factor prox-1 in patients with CD, was associated with a higher risk of postoperative recurrence [111,112]. Furthermore, in a murine model, it was found that platelets can migrate to lymphatic vessels, suppress lymphangiogenesis and exacerbate colonic inflammation by inhibiting the clearance of immune cells [113].

Several novel therapeutic interventions interfering with lymphangiogenesis have been studied in DSS or IL-10 KO colitis mouse models. VEGFC-promoted lymphangiogenesis reduced inflammation. Similarly, blocking VEGFR-3 or the calcitonin lymphangiogenic receptors resulted in disease exacerbation [114,115,116,117,118,119]. On the contrary, it was shown that the pharmacological inhibition of lymphangiogenesis ameliorated disease activity [120,121], while lymphangiogenesis stimulation by VEGF-C overexepression in the same colitis model aggravated intestinal inflammation [122]. Overall, it is unclear whether lymphangiogenesis promotes or suppresses inflammatory responses in IBD and more human studies are warranted.

### 2.4. Granulomas in Mesenteric Lymph Nodes and Lymphatic Vessels as Indication of Crohn’s Disease

As lymphatic vessels and endothelium have a role in IBD pathogenesis, it is of importance to consider the potential role of obstruction of the GALT lymphatics in regulating disease activity. Lymphatic obstruction is often caused by the presence granulomas in MLNs of patients with CD [2,10,123]. In cohorts of patients with CD who underwent ileocolonic resection, only patients with MLN granulomas were associated with higher risk of both postoperative, endoscopic and surgical recurrence [124,125].

The cellular composition of these granulomas might also be of significance for the CD pathogenetic mechanisms, as well as, for diagnostic purposes. In patients with CD with granulomatous lymphadenitis, it was shown that granulomas in MLNs were composed of T-helper cells and monocyte/macrophage derived epithelioid cells with characteristics of antigen-presenting cells (APCs) [126]. It was also observed that granulomas in draining LNs were associated with B-cell zone hyperplasia [66]. In addition, lymphatic vessel granulomas in patients with CD (complicated with granulomatous lymphangitis) were composed of epithelioid cells [127,128,129]. Therefore, the cellular characteristics of granulomas might be a useful tool for the detection and classification of CD comorbidities, although further studies are needed to substantiate this.

### 2.5. The Interaction between the Microbiome and the Lymphatic System in IBD

There is conflicting evidence concerning bacterial translocation to lymph nodes in patients with CD [11,130]. In mice, the intestinal microbiota inhibits the trafficking of both commensal and pathogenic bacteria to MLNs, whereas in conditions of dysbiosis (which is often associated with IBD), non-invasive bacteria colonized the MLNs. This MLN bacterial colonization was also associated with CCR7 chemokine receptor dependent trafficking of CX_3_CR1^+^ mononuclear phagocytes to MLNs [12]. Moreover, when colitis mice were treated with the antibiotic rifaximin, bacterial translocation to MLNs was inhibited and the histological score of the disease improved [131]. From this perspective, targeting bacterial trafficking could be exploited therapeutically. Conclusively, dysbiosis in MLNs might have a role in IBD pathogenesis, and restoring it by disrupting bacterial translocation to MLNs might be a novel potential therapeutic approach.

Finally, in a comparative study, a significantly distinct microbial flora composition was identified between the MLNs of patients with UCand CD. In patients with CD, the MLN microbiome is characterized by reduced species diversity [132]. Therefore, the distinctive MLN microbiota in UC and CD could be a novel differential diagnostic tool in patients with an indeterminate colitis at resection. However, follow-up studies are essential to validate this finding before it can be applied in clinical practice.

### 2.6. Lymphocyte Trafficking as a Potential Target for IBD Treatment

As lymphoid tissue clearly plays a role in driving the immune responses in IBD and lymphocyte trafficking between GALT compartments is a key feature, the disruption of this process might be used as a therapeutic intervention.

There were efforts to target specific chemokines to interfere with the lymphocyte trafficking process. For instance, an antagonist of the CCR9 chemokine receptor called vercinon was tested in a phase 3 clinical trial in patients with CD, but did not demonstrate significant benefit [133]. Furthermore, in a phase 2 clinical trial with patients with UC, eldemumab, a monoclonal antibody blocking the CXCL-10 chemokine, failed to induce clinical remission [134].

Currently, there are two effective approaches to inhibit lymphocyte trafficking: 1. blocking adhesion molecules and 2. targeting the Sphingosine-1 phosphate receptor family.

#### 2.6.1. Adhesion Molecule Targeting as a Treatment Option

Targeting leukocyte infiltration in the colonic mucosa by blocking adhesion molecules proved to be effective in experimental colitis models [135,136,137]. One of the first indications that inhibiting lymphocyte trafficking could work as a potential treatment for IBD was observed when the oral administration of saccharomyces (S) boulardi attenuated colitis in mice. The treatment with S boulardii trapped Th1 cells in MLNs by enhancing their adhesiveness towards lymphatic endothelial cells through upregulation of integrins and selectins, hence, preventing their infiltration in the inflamed colon [138].

The first monoclonal antibody against the a4 integrin (α4β1 and α4β7) that was approved for CD is natalizumab; however, its use is limited because it is linked with the development of progressive multifocal leukoencephalopathy (PML), a fatal nervous system infection [139]. The targeting of adhesion molecule a4β7 using the monoclonal antibody vedolizumab also proved to be an efficient therapeutic strategy in IBD, but without confirmed PML cases as a serious complication [13,140,141,142,143,144]. An interesting observation was that vedolizumab treatment altered the M1/M2 macrophage ratio but did not alter T-lymphocyte recruitment in the affected areas of the gut [145]. Another comparable monoclonal antibody is etrolizumab, which targets the beta 7 subunit of a4β7 and aEβ7 integrins [146]. Using etrolizumab for the treatment of mice with DSS-induced colitis was efficient in reducing mucosal inflammation and inhibited colonic recruitment of CD8^+^ T-cells and Th9 cells [147]. Clinical trials in patients with IBD are currently ongoing [148]. Moreover, the inhibition of the non-integrin CD151 molecule, which forms a complex with multiple integrins in T cells, resulted in reduced colitis progression in mice. This blocking peptide offers an alternative potential therapeutic strategy [149].

Using small molecules instead of antibodies to target adhesion molecules is a potential therapeutic option. A synthetic oral antagonist against a4 integrin has been shown to reduce colitis severity in mice by inhibiting lymphocyte homing in PPs and T-cell infiltration in the lamina propria [150]. This a4 integrin antagonist could also induce clinical remission in patients with UC but not with CD [151,152]. Furthermore, alicaforsen, a synthetic antisense oligonucleotide against the adhesion molecule ICAM-1, proved to be effective in patients with UC in phase 2 trials and especially in patients with UC-related pouchitis in phase 3 trials [153,154,155,156,157,158,159,160], whereas in patients with CD, alicaforsen did not significantly improve the clinical outcome [161,162,163,164,165]. Additionally, it was observed that a tellurium-based compound could preserve the colonic epithelium integrity in the DSS colitis mouse model by preventing the migration of a4β7^+^ macrophages to the colon and the adhesion of MLN cells to MadCAM-1 both in vitro and in vivo. This compound also reduced the CD4^+^ T-cell colonic infiltration and increased the T-reg accumulation in the colon, but the mechanism of action behind this effect is unclear [166].

#### 2.6.2. Targeting S1P Receptor as a Novel Therapeutic Approach

Another approach to alter lymphocyte trafficking is by modulating the Sphingosine-1 phosphate (S1P) receptor. There are 5 S1P receptor subtypes and targeting S1P1 receptor could inhibit the migration of lymphocytes from thymus and secondary peripheral lymphoid organs [167,168]. A non-selective S1P receptor modulator, called fingolimod, which disrupts lymphocyte trafficking by trapping the CCR7^+^ T cells in LNs is already used as a therapeutic agent in multiple sclerosis [168,169]. The same agent was tested on several experimental colitis models and was found to improve disease progression [170,171,172,173]. S1P receptor targeting in a chronic ileitis mouse model altered the trafficking of T-cells between MLNs and peripheral blood by inducing the degradation of the receptor in lymphocytes and endothelial cells, and ameliorated disease activity [174,175].

Ozanimod is another therapeutic agent that targets specifically the S1P1/5 receptor subtypes. Ozanimod was shown to induce the internalization and degradation of these receptors, improve the histological score in mice with TNBS (and adoptive CD4^+^CD45RB^+^T-cell transfer mediated) colitis and reduce the numbers of circulating B and CCR7^+^ T-lymphocytes [176]. Ozanimod also proved to be effective in phase 2 clinical studies in patients with CD, as well as in phase 3 studies with patients with UC, and has recently received market approval for UC [14,177]. Another compound that targets S1P1/3/5 receptors, etrasimod, attenuated disease activity in the CD4^+^CD45RB^+^T-cell adoptive transfer colitis mouse model [178] and demonstrated therapeutic efficacy in a phase 2 clinical trial of patients with UC [179]. Amiselimod, a novel, more selective S1P1 receptor-modulating compound improved the clinical and histological score of colitis in a CD4^+^CD45RB^+^ T-lymphocyte adoptive transfer model by reducing the infiltration of Th1 and Th17 cells in the colonic mucosa. This compound demonstrated comparable efficacy with anti-TNFα monoclonal antibodies in the same mouse model [180]. However, a recent phase 2 study did not show therapeutic benefit in patients with CD [181]. Overall, these results indicate that lymphocyte trafficking disruption, through the S1P pathway manipulation by targeting the S1P1 receptor, could be of major significance for IBD drug development.

## 3. Discussion

There is extensive evidence supporting the involvement of the lymphatic system in IBD pathophysiology. The interaction between innate and adaptive immune responses in MLNs and PPs can lead to Th1 and Th17 polarization, which is linked to the disease onset. Therapeutic targeting of the Th1/Th17 differentiation pathways has been proven successful in animal models of the disease [22,36,40,41,44,45,46,47,48,49,50,51]. However, it should be mentioned that, in these studies, most IBD mouse models were TNBS and DSS induced, which resemble human UC more than CD. In addition, lymphocyte trafficking plays an important role in IBD pathophysiology [56,57,58,59]. Disrupting this process by targeting adhesion molecules or the S1P receptor appears to be an effective therapeutic strategy and more clinical trials are ongoing in this direction [13,148,176,177,178,179,182,183]. Additional studies are mandatory to further clarify the exact role of T-cell polarization and lymphocyte trafficking in various stages and subtypes of the disease. Moreover, studies involving in-depth molecular characterization of target tissues before and after treatment with lymphocyte trafficking inhibitors could further unravel their exact therapeutic mechanism and could lead to optimization of the current treatment protocols.

Limited data are available on MLN and PPs in humoral responses, as well as on the implication of lymph node stroma in IBD pathophysiology [67,68,71,73,83,84,90,91,94]. While lymphatic obstruction by granulomas in MLNs and lymphatic vessels correlates with disease severity [2,10,123,124,125], data on the role of lymphangiogenesis in disease activity are contradictory [116,117,120,121,122]. Further studies are essential to better understand the involvement of these processes in IBD pathology. In addition to that, alterations in the MLN microbiome, such as MLN dysbiosis, could possibly trigger the immune deregulation observed in IBD [11,12]. Potentially, the MLN microbiome can be manipulated therapeutically by inhibition of MLN bacterial trafficking or by restoring dysbiosis [131].

In conclusion, the lymphatic system is implicated in the initiation and progression of IBD, but to gain a better insight in the exact underlying immune mediated inflammatory disease mechanism, further research not only in animal models of IBD but also in (tissues of) patients with IBD is required. To date, most studies used animal models to investigate the lymphatic system in IBD. The recent development of the inguinal LN biopsy procedure as a research tool in the context of rheumatoid arthritis [184] opens up possibilities to investigate peripheral LNs in IBD and conduct comparative studies between peripheral LNs and MLNs in humans. By studying peripheral LNs, it is possible to further explore the role of the lymphatic system in the immunopathogenesis of IBD. Furthermore, it allows the possibility to conduct studies investigating the mechanisms driving the therapeutic effect of lymphocyte trafficking inhibitors.

## 4. Materials and Methods

A systematic search in PubMed (from its inception to November of 2021) focusing on components of the lymphatic system in IBD was conducted. The concepts for our literature search were based on the following general research questions:

1. What is the role of the adaptive and innate immune cells, as well as, stroma (including lymphatic endothelium) located in lymphoid tissues, regarding the pathogenesis of IBD (Crohn’s disease or ulcerative colitis)?
2. What is the role of lymphatic obstruction in disease initiation and progression?
3. Is there an interaction between the lymphoid tissues and the microbiome in relation to IBD pathogenesis?
4. Which are the treatment options and targets interfering with lymphocyte trafficking in the GALT?

**Figure 3:**
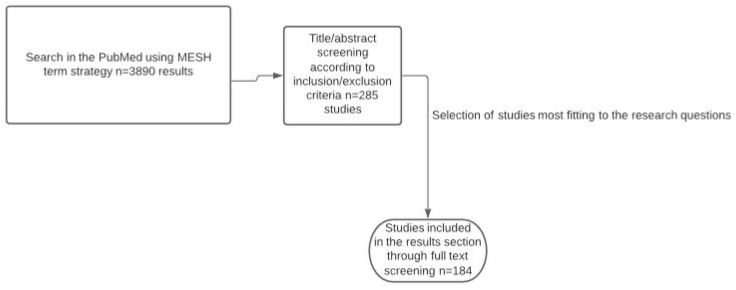
Study selection diagram.

To answer these research questions, we followed a single search strategy with mesh terms in PubMed focused on the GALT compartments and peripheral lymphoid tissues in the context of IBD (Figure 3) (see Appendix A for full search details). Abstracts from conference proceedings were not included and review articles were used to identify additional eligible studies.

### Inclusion and Exclusion Criteria

Studies were included if they reported on lymphoid tissues related to Crohn’s disease patients, AND/OR ulcerative colitis patients, AND/OR rodent animal models of IBD. Language restrictions were not applied. Articles related to non-English literature were checked by the English version abstracts in PubMed. We included all studies that had an abstract/full text publicly available and were relevant to our research questions.

We excluded studies in which IBD was correlated with any kind of neoplasm or infection, since these conditions could have affected the lymphatic compartment alterations observed in these studies. All non-rodent animal models of IBD were excluded. We did not search or include studies that reported on bone marrow, thymus or spleen because they are beyond the scope of our research questions.

## Data Availability

Not applicable.

## Appendix A

We searched PubMed with the following search query:

((“granuloma”[MeSH Terms] OR granuloma*[tiab]) OR (“lymphatic vessels”[MeSH Terms] OR lymphatic-vessels*[tiab] or lymphatic-system[tiab] or lymphatics[tiab]) or (“lymph nodes”[MeSH Terms] OR lymph-node*[tiab] or lymph[tiab]) or ((“lymphoid tissue”[MeSH Terms] OR lymphoid-tissue*[tiab] or “lymphocytes”[MeSH Terms] OR “lymphocyte count”[MeSH Terms] OR lymphocytes[tiab]) AND traffic*[tiab])) AND (“Inflammatory Bowel Diseases” [Mesh] OR “Crohn*”[tiab] OR “ulcerative colitis”[tiab]).
